# The Role of Physical Activity and Physical Function in Predicting Physical Frailty Transitions in Chinese Older Adults: Longitudinal Observational Study From CHARLS

**DOI:** 10.2196/75887

**Published:** 2025-09-15

**Authors:** Ziwei Zeng, Chun Liang Hsu, Cindy Hui-ping Sit, Stephen Heung-sang Wong, Yijian Yang

**Affiliations:** 1 Department of Sports Science and Physical Education, The Chinese University of Hong Kong, Shatin, Hong Kong, China (Hong Kong), 852 39434001; 2 Department of Rehabilitation Sciences, The Hong Kong Polytechnic University, Hong Kong, China (Hong Kong); 3 The Jockey Club Institute of Ageing, The Chinese University of Hong Kong, Hong Kong, China (Hong Kong)

**Keywords:** elderly, frailty trajectories, physical performance, strength, mobility

## Abstract

**Background:**

Frailty is a dynamic geriatric syndrome associated with adverse health outcomes, yet its progression can be mitigated through targeted interventions.

**Objective:**

This study aimed to investigate predictors of frailty transitions in Chinese older adults, focusing on physical activity (PA) and physical function.

**Methods:**

Using data from the China Health and Retirement Longitudinal Study (CHARLS), we examined transitions between frailty states (robust, prefrail, and frail) from 2011 (baseline) to 2013 (follow-up) among 1014 participants aged 65 years and older. The following outcomes were assessed, including frailty using the physical frailty phenotype, PA using a modified International Physical Activity Questionnaire, and physical function using the Short Physical Performance Battery (SPPB) and handgrip strength. Ordinal logistic regression models were used to examine the relationship between PA, physical function, and frailty transitions.

**Results:**

Results showed that higher PA levels and better physical function reduced the likelihood of worsening frailty or increased the probability of transitioning to robustness. Key findings from the subgroup include: among robust individuals, greater handgrip strength predicted maintained robustness (average marginal effects [AME]=1.12%; *P*=.02); in prefrail individuals, higher vigorous PA (AME=21.76%; *P*=.04) and handgrip strength (AME=0.64%; *P*=.003) increased transitions to robustness; for frail individuals, increased low-intensity PA (AME =22.48%; *P*=.04) and higher SPPB walking subscores (AME=27.73%; *P*=.02) promoted improvement to nonfrailty.

**Conclusions:**

These findings highlight the importance of tailored interventions based on baseline frailty status. Promoting PA and improving physical function, particularly muscle strength and mobility function, may help delay or reverse frailty progression.

## Introduction

The global population is aging at an unprecedented rate, presenting significant challenges to public health and social care systems worldwide [1]. By 2050, the proportion of adults aged 65 years and older in China is projected to reach 26%, reflecting a broader demographic shift that underscores the urgency of addressing age-related health issues such as physical frailty [2]. Frailty, a geriatric syndrome characterized by diminished physiological reserves and increased vulnerability to stressors, is associated with adverse outcomes including falls, disability, hospitalization, and mortality [3,4]. Defined by Fried et al [3] as the presence of 3 or more criteria: weakness, slow gait speed, exhaustion, low activity, and unintentional weight loss, physical frailty is a dynamic condition that is not an inevitable consequence of aging but rather a potentially reversible state [3,5]. Rates of physical frailty among Chinese older adults range from 6% to 17% [6], highlighting the importance of early detection and intervention to mitigate its progression.

Frailty is typically classified into 3 stages: robust, prefrail, and frail, with individuals transitioning between these stages over time due to a combination of physical, psychological, and environmental factors [7]. Understanding these transitions is crucial for developing targeted interventions to prevent or delay frailty progression. Physical function measures, such as handgrip strength and the Short Physical Performance Battery (SPPB), are increasingly recognized as critical indicators of frailty [8-11]. Handgrip strength, for example, provides insight into muscular strength, functional limitations, and the onset of age-related adverse health conditions, making it a more useful marker of frailty than chronological age alone [8,9]. Similarly, the SPPB, which assesses gait speed, sit-to-stand (STS) performance, and balance, has been widely used to evaluate physical function and predict frailty progression [10-12]. These measures offer a comprehensive understanding of the factors that influence frailty dynamics and complement the role of physical activity (PA) in frailty transitions [13].

PA has long been recognized as a cornerstone of healthy aging, encompassing any bodily movement produced by skeletal muscles that increases energy expenditure [14]. Beyond its general health benefits, PA has emerged as a key modifiable factor in frailty prevention and management [15]. Regular PA has been shown to improve muscle strength, cardiovascular health, and overall physical function, all of which are critical for reducing the risk of frailty [16-22]. However, the specific types and intensities of PA that influence frailty transitions remain unclear, highlighting the need for further investigation.

In China, the rapid aging population presents challenges in addressing the burden of frailty and functional decline [23]. Despite the growing recognition of frailty as a public health issue, few studies have examined the specific factors that predict frailty transitions among Chinese older adults. Existing research has primarily focused on Western populations [24,25], and there is limited evidence on how PA and physical function influence frailty transitions in the Chinese context. This gap underscores the need for population-specific research, as contextual factors (eg, lifestyle and health care infrastructure [26]) in China may differ from Western settings. Furthermore, longitudinal follow-up is critical for understanding frailty as a dynamic process rather than a static condition. By observing the same individuals over time, researchers can quantify both progression and recovery across frailty states and relate these transitions to changes in PA and physical function. This design overcomes the limitations of cross-sectional studies where reverse causation and unmeasured baseline differences may obscure causal pathways and provides more robust evidence to inform stage-specific interventions. Understanding these relationships is essential for developing tailored strategies to mitigate frailty in Chinese older adults.

This study aimed to investigate the predictors of physical frailty transitions in Chinese older adults, with a particular focus on the role of PA participation and physical function. We hypothesized that higher amounts of light, moderate, and vigorous PA, along with greater muscle strength and mobility, would increase the likelihood of transitioning to a more robust status. Conversely, lower amounts of PA across intensity categories and poor physical function would increase the probability of worsening frailty. By examining these relationships, this study provided valuable insights into the role of PA and physical function in physical frailty transitions, with the ultimate goal of informing intervention strategies to delay or prevent frailty progression.

## Methods

### Study Design

This was a 2-wave longitudinal observational study using data from the China Health and Retirement Longitudinal Study (CHARLS). We leveraged the baseline survey (Wave 1, 2011) and first follow-up (Wave 2, 2013) to examine within-person transitions among 3 frailty states (robust, prefrail, and frail), and to evaluate how baseline PA and physical function predicted these transitions.

### Ethical Considerations

Data were obtained from CHARLS, a nationally representative survey designed to examine the aging process among Chinese households and individuals aged 45 years and older [27]. The sampling design used a 4-stage, stratified, cluster method, covering 150 county-level and 450 village-level units across China [28]. Ethical approval was granted by the ethical review committee of Peking University (approval IRB00001052-11015), and all participants provided written informed consent. CHARLS adheres to the declaration of Helsinki and China’s Personal Information Protection Law. The CHARLS database ensures strict privacy protection and anonymization during data collection and processing to safeguard participants’ personal information.

### Study Participants

The CHARLS survey collected comprehensive data on demographics, anthropometrics, health status, functioning, and physical measurements [27]. This study used data from the first 2 waves of CHARLS (2011 and 2013), which share identical sampling procedures and therefore draw from the same population base. Although frailty is typically a slow-progressing condition, previous longitudinal research suggests that meaningful changes, both improvements and declines, can be captured within a 2-year period among older adults [29,30]. Thus, the Wave 1 (2011) and Wave 2 (2013) data are appropriate for examining frailty transitions in this context.

While the Law of the People’s Republic of China on the Protection of the Rights and Interests of the Elderly (1996) defines older adults as individuals aged  60  years and older, we adopted the United Nations’ recommendation of  ages  65  years and older. This definition aligns with international standards in frailty research and better targets the population at higher risk for physical frailty in China [31].

For this study, we first restricted the sample to respondents who completed both Wave 1 and Wave 2 data collection. From this sample, we included participants who met the following criteria: (1) participants aged 65 years and older at baseline (2011); (2) they provided sufficient data on physical frailty (at least 3 components), PA, and physical function (at least 1 component of the SPPB) across both waves; and (3) had consistent and valid data for gender, body height, and body weight across both waves (eg, mismatched gender data between waves were excluded).

### Measures

#### Physical Frailty

Physical frailty was assessed using the Physical Frailty Phenotype (PFP) scale, developed from the Cardiovascular Health Study (CHS) [3]. The scale comprises five criteria: (1) weakness, (2) slowness, (3) exhaustion, (4) low activity, and (5) shrinking. The PFP scale was adapted and validated for use in the CHARLS to examine physical frailty in older Chinese adults [32-35]. Each criterion was scored as 1 point if met, with a total score ranging from 0 to 5. Participants were categorized as “Robust” (0 criteria met), “Pre-frail” (1‐2 criteria met), or “Frail” (3‐5 criteria met) [36]. Detailed definitions and scoring for each criterion are provided in Table 1.

**Table 1.** Operationalization of the Physical Frailty Phenotype in the China Health and Retirement Longitudinal Study (CHARLS) compared to the Cardiovascular Health Study (CHS).

PFP^b^ component	CHS^c^ definition	CHARLS^a^ adaptation		
		Definition	Values	
			Women	Men
Weakness	Handgrip strength: lowest 20% (by gender and BMI).	Handgrip strength	BMI ≤20.6kg/m^2^: ≤18.0kg 20.6<BMI≤23.1kg/m^2^:≤18.2kg 23.1<BMI ≤25.7kg/m^2^: ≤20.0kg BMI >25.7kg/m^2^: ≤20.0kg	BMI ≤20.0kg/m^2^: ≤27.0kg 20.0<BMI≤22.0kg/m^2^: ≤28.5kg 22.0<BMI ≤24.4kg/m^2^: ≤30.0kg BMI >24.4kg/m^2^: ≤31.5kg
Slowness	Walking time/15 feet: slowest 20% (by gender and height).	Walking speed (2.5 meters)	Body height ≤151cm: ≤0.41m/s Body height >151cm: ≤0.45m/s	Body height ≤162cm: ≤0.47m/s Body height >162cm: ≤0.50m/s
Exhaustion	“Exhaustion” (self-report).	Self-reported	Felt “I felt everything I did was an effort” or “I could not get going” about 3‐4 days or 5‐7 days in a week.	
Low activity	Kcals/week: lowest 20%: men:<383 Kcals/week; women:<270 Kcals/week.	Self-reported	Answered “no” to: “During a usual week, did you do any vigorous activities/moderate physical effort/walking for at least ten minutes continuously.”	
Shrinking	Baseline: >10 lbs lost unintentionally in the previous year.	Self-reported	Lost 5 or more kilograms in the last year in Wave 1 (2011). Weight declines 5 or more kilograms between Wave 1 (2011) and Wave 2 (2013).	

a CHARLS China Health and Retirement Longitudinal Study.

b PFP Physical Frailty Phenotype.

c CHS Cardiovascular Health Study.

Initially, mortality was considered a key transition outcome, but upon further review, it was found that participants who had died by Wave 2 did not provide sufficient baseline data for inclusion. Therefore, mortality was not considered as a transition outcome in this study. Instead, we focused on the 3 states of physical frailty (robust, prefrail, and frail). Frailty transitions were defined as movements between these three states over time, categorized as follows: remain robust, robust to prefrail, robust to frail, prefrail to robust, remain prefrail, prefrail to frail, frail to robust, frail to prefrail, and remain frail.

#### Physical Activity

PA was assessed using a modified version of the International Physical Activity Questionnaire (IPAQ), a widely used tool for PA assessment [27]. Differences between CHARLS and the original IPAQ included: (1) PA was assessed over a “usual week” rather than the “last 7 days,” (2) sedentary behavior was not recorded, and (3) PA was reported as discrete time categories rather than continuous data [37]. PA data included intensity (vigorous, moderate, and low-intensity), duration (<30 min, 30 min–2 h, 2‐4 h, and ≥4 h), and frequency (1‐7 d/wk). PA volume was calculated using metabolic equivalents (METs): vigorous-intensity=8.0 METs, moderate-intensity=4.0 METs, and low-intensity (walking)=3.3 METs [37,38]. Vigorous PA MET-minutes/week=8.0×vigorous-intensity activity minutes×vigorous-intensity days; Moderate PA MET-minutes/week=4.0×moderate-intensity activity minutes × moderate-intensity days; and low-intensity PA MET-minutes/week=3.3×walking minutes×walking days [37,38]. Total PA was categorized into four types based on MET-minutes/week for each intensity level: (1) null type=0, (2) type 1: <600, (3) type 2: 600‐3000, and (4) type 3: >3000, following the IPAQ scoring protocol and previously applied in similar Chinese cohorts [37,39].

#### Physical Function

Physical function was assessed using the SPPB, which includes tasks measuring gait speed, STS test time, and standing balance. Implementation details and scoring for the Chinese population were described by Zhong et al [40]. Each test is scored from 0 to 4, with higher scores indicating better performance. The total SPPB score (range 0‐12) was classified into three groups: 0‐6 (poor performers), 7‐9 (fair performers), and 10‐12 (good performers) [41].

Handgrip strength was assessed using a handheld dynamometer (Yuejian WL-1000; Nantong Yuejian Physical Measurement Instrument Co Ltd). Participants were instructed to stand upright, maintain a 90° elbow flexion, and perform maximal grip exertion for 3‐5 seconds. A total of 2 trials were conducted on each hand, and the highest value (kg) across all 4 trials was used for analysis [27].

### Covariates

Covariates were selected based on their relevance to physical frailty, PA, and physical function. These included age, gender (man=0 and woman=1), BMI, marital status (unmarried=0 and married=1), education level (illiterate=1, no formal education=2, elementary school=3, and middle school or above=4), self-rated health (poor or fair=0 and good or very good or excellent=1), fall history (no=0 and yes=1), activity of daily living (ADL) limitation (without=0 and with=1), instrumental activity of daily living (IADL) limitation (without=0 and with=1), number of chronic diseases (0=0, 1=1, and ≥2=2), pain status (no=0 and yes=1), current smoking status (no=0 and yes=1), and current drinking status (no=0 and yes=1).

### Statistical Analysis

Data analysis was conducted using Stata 15.0 (StataCorp LP), with statistical significance set at *P*<.05. Descriptive statistics (mean [SD] for continuous variables and n [%] for categorical variables) were used to summarize baseline characteristics. Multiple imputation by chained equations addressed missing data, with results combined across 20 imputations [42]. Differences across frailty groups at baseline were examined using the 1-way ANOVA test (normally distributed data) or the Kruskal-Wallis H test (non-normally distributed data) for continuous variables, and *χ*^2^ tests for categorical variables. Post hoc pairwise comparisons were performed with Bonferroni corrections as appropriate.

Ordinal logistic regression was used to model the relationship between predictors (PA and physical function) and the ordered outcome of frailty transitions, adjusting for demographic and health-related covariates [43]. Average marginal effects (AMEs) were calculated to assess the impact of a 1-unit increase in a predictor on the probability of frailty transition state, when holding other variables constant. To assess potential circularity from including handgrip strength (one frailty criterion) as a predictor, we conducted a sensitivity analysis omitting handgrip strength from the model. Robust standard errors were used to account for potential heteroskedasticity. Predictive accuracy was assessed using a classification table, and discriminate ability (remain frail or worsen to frailty vs others) was evaluated using receiver operating characteristic (ROC) curves and the area under the curve (AUC) was calculated along with corresponding 95% CIs and *P* values to assess statistical significance [44]. Separate ROC curves were generated for each baseline frailty status.

## Results

### Sample Characteristics and Comparisons

**Figure 1.**
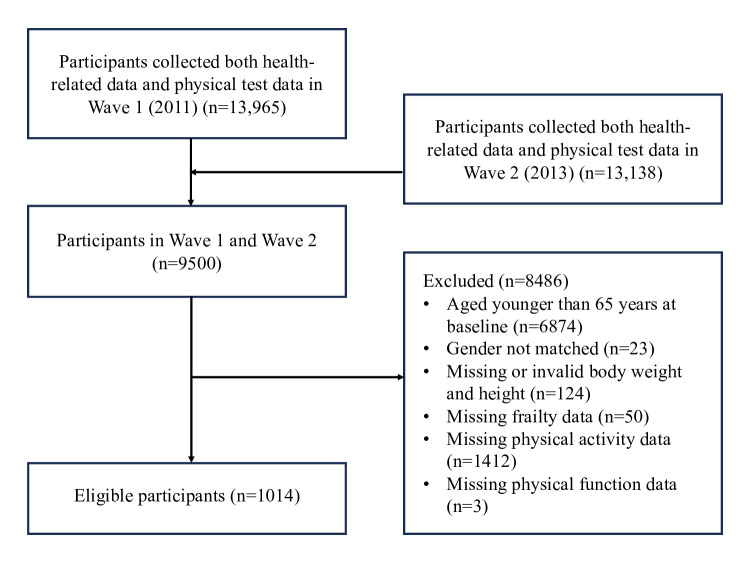
Flowchart of participant selection.

**Figure 2.**
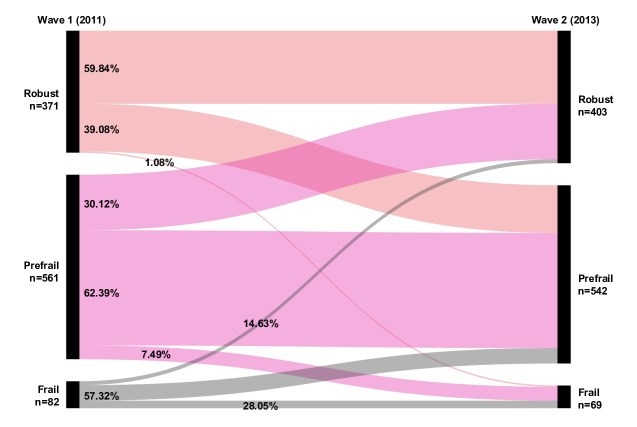
Sankey diagram of frailty status transition from Wave 1 (2011) to Wave 2 (2013).

A total of 1014 participants were included (see Figure 1), with 371 categorized as robust, 561 as prefrail, and 82 as frail at baseline. Figure 2 shows the transition patterns from baseline to follow-up. Among robust participants, 39% (145/371) transitioned to prefrailty, and 1% (4/371) transitioned to frailty. Among prefrail participants, 7% (42/561) became frail, while 30% (169/561) reverted to a robust state. Among frail participants, 15% (12/82) regained robustness, and 57% (47/82) became prefrail.

The mean age of the study population was 71.1 years (SD 5.0, range 65‐90 y), with significant variations across frailty groups (robust: 70.2 y [SD 4.4], prefrail: 71.4 y [SD 5.1], and frail: 73.0 y [SD 5.8]; *P*<.001). Gender distribution was balanced (48% women). Frailer participants were significantly older, had lower educational attachment, poorer self-rated health, higher fall history, and greater limitations in ADL and IADL, as well as more pain and chronic diseases compared to their healthier counterparts (*P*≤.01). Detailed baseline characteristics are presented in Table 2.

**Table 2.** Demographic characteristics of participants by baseline frailty status.

Variables	Total sample (N=1014)	Robust (n=371)	Prefrail (n=561)	Frail (n=82)	*P* value
Age (years), n (%)					<.001^a^
65‐74	775 (76.43)	312 (84.10)	414 (73.80)	49 (59.76)	
75‐84	229 (22.58)	58 (15.63)	141 (25.13)	30 (36.59)	
≥85	10 (0.99)	1 (0.27)	6 (1.07)	3 (3.66)	
Gender, n (%)					.02^a^
Women	486 (47.93)	156 (42.05)	286 (50.98)	44 (53.66)	
Men	528 (52.07)	215 (57.95)	275 (49.02)	38 (46.34)	
BMI (kg/m^2^), mean (SD)	22.37 (3.57)	22.65 (3.64)	22.14 (3.47)	22.66 (3.89)	.09
Marital status, n (%)					.91
Unmarried	253 (24.95)	91 (24.53)	140 (25.96)	22 (26.83)	
Married	761 (75.05)	280 (75.47)	421 (75.04)	60 (73.17)	
Education level, n (%)					.004^a^
Illiterate	392 (38.66)	123 (33.15)	226 (40.29)	43 (52.44)	
Primary and lower	201 (19.82)	69 (18.60)	119 (21.21)	13 (15.85)	
Secondary education	237 (23.37)	93 (25.07)	130 (23.17)	14 (17.07)	
College and above	184 (18.15)	86 (23.18)	86 (15.33)	12 (14.63)	
Self-rated health, n (%)					<.001^a^
Poor or fair	825 (81.36)	268 (72.24)	481 (85.74)	76 (92.68)	
Good or very good or excellent	189 (18.64)	103 (27.76)	80 (14.26)	6 (7.32)	
Fall history, n (%)					.01^a^
Yes	176 (17.36)	47 (12.67)	111 (19.79)	18 (21.95)	
No	838 (82.64)	324 (87.33)	450 (80.21)	64 (78.05)	
ADL^b^ limitation, n (%)					<.001^a^
With	261 (25.74)	58 (15.63)	160 (28.52)	43 (52.44)	
Without	753 (74.26)	313 (84.37)	401 (71.48)	39 (47.56)	
IADL^c^ limitation, n (%)					<.001^a^
With	291 (28.70)	60 (16.17)	178 (31.73)	53 (64.63)	
Without	723 (71.30)	311 (83.83)	383 (68.27)	29 (35.37)	
Number of chronic diseases, n (%)					.007^a^
0	270 (26.63)	118 (31.81)	141 (25.13)	11 (13.41)	
1	285 (28.11)	102 (27.49)	159 (28.34)	24 (29.27)	
≥2	459 (45.27)	151 (40.70)	261 (46.52)	47 (57.32)	
Pain, n (%)					<.001^a^
Yes	358 (35.31)	80 (21.56)	232 (41.35)	46 (56.10)	
No	656 (64.69)	291 (78.44)	329 (58.65)	36 (43.90)	
Current smoking, n (%)					.51
Yes	423 (41.72)	162 (43.67)	225 (40.11)	36 (43.90)	
No	591 (58.28)	209 (56.33)	336 (59.89)	46 (56.10)	
Current drinking, n (%)					.02^a^
Yes	306 (30.18)	129 (34.77)	160 (28.52)	17 (20.73)	
No	708 (69.18)	242 (65.23)	401 (71.48)	65 (79.27)	
SPPB^d^ total score (points), mean (SD)	8.92 (2.11)	9.84 (1.68)	8.66 (2.06)	6.55 (1.89)	<.001^a^
SPPB-Walk (points), mean (SD)	2.47 (0.99)	2.82 (0.85)	2.38 (1.00)	1.46 (0.72)	<.001^a^
SPPB-STS^e^ (points), mean (SD)	2.98 (1.16)	3.37 (0.93)	2.86 (1.18)	2.01 (1.20)	<.001^a^
SPPB-Balance (points), mean (SD)	3.48 (0.81)	3.64 (0.68)	3.42 (0.84)	3.07 (0.91)	<.001^a^

a Indicates statistical significance (*P*<.05).

b ADL: activity of daily living.

c IADL: instrumental activity of daily living.

d SPPB: Short Physical Performance Battery.

e STS: sit-to-stand.

**Figure 3.**
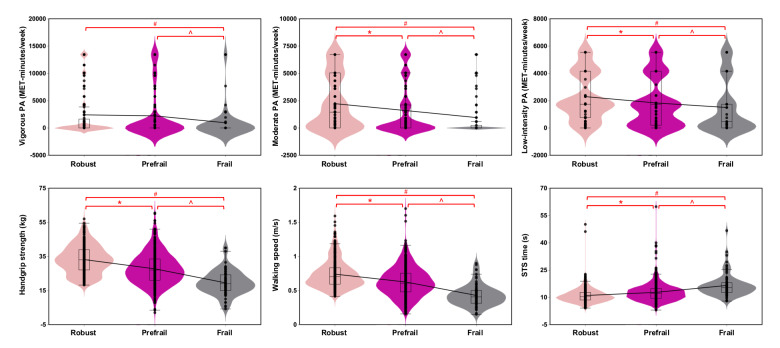
Physical activity participation and physical function across physical frailty groups at baseline. The “*” sign denotes significant differences between robust and pre-frail; “^” denotes significant differences between prefrail and frail; “#” denotes significant differences between robust and frail. The shape of the violin plot represents a kernel density estimate, illustrating the distribution of data across different values. The wider section of the violin indicates a denser data distribution. The thick line in the center of the violin marks the median, while the box plot shows the IQR and outliers. MET: metabolic equivalent; PA: physical activity; STS: sit-to-stand.

Table 2 and Figure 3 show that robust participants engaged in significantly more moderate and low-intensity PA compared to pre-frail and frail individuals (*P*<.001), with vigorous PA significantly higher in robust individuals compared to frail (*P*=.003). Prefrail participants also demonstrated higher PA levels compared to frail individuals (*P*≤.02). Robust participants exhibited greater handgrip strength, faster walking speeds, shorter STS time, and higher SPPB scores compared to prefrail participants, and prefrail participants performed better than frail participants (*P*<.001)

### Predictors of Frailty Transitions

**Figure 4.**
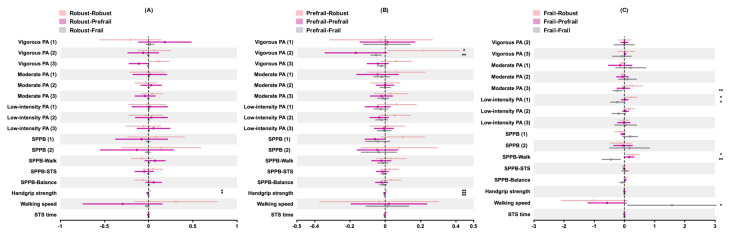
Average marginal effect (AME) of each predictor on the probability of frailty transition in older adults. (**A**) “Robust” at baseline; (**B**) “Pre-frail” at baseline; (**C**) “Frail” at baseline. Notes: AME reflects the change in the probability of each frailty transition at follow-up based on a 1-unit change in a predictor. The “*” sign denotes *P*<.05 and “**” denotes *P*<.01. PA: physical activity; SPPB: Short Physical Performance Battery; STS: sit-to-stand.

For participants robust at baseline, increased handgrip strength (AME=1.12%, 95% CI 0.21-2.03; *P*=.02) was associated with a higher probability of remaining robust, while decreased handgrip strength (AME=−1.06%, 95% CI −1.93 to −0.19; *P*=.02) was linked to a higher probability of transitioning to prefrailty (see Figure 4A).

Among prefrail participants, increased participation in vigorous PA (type 2; AME=21.76%, 95% CI 1.31%-42.20%; *P*=.04) and better handgrip strength (AME=0.64%, 95% CI 0.21%-1.07%; *P*=.003) were associated with a greater probability of transitioning to robust state. Conversely, decreased vigorous PA (type 2) participation (AME=−5.12%, 95% CI −8.38% to −1.87%; *P*=.002) and handgrip strength (AME=−0.23%, 95% CI −0.40% to −0.07%; *P*=.005) were linked to a higher likelihood of worsening to frailty. Furthermore, decreased handgrip strength (AME=−0.41%, 95% CI −0.70% to −0.13%; *P*=.005) also increased the probability of remaining prefrail (see Figure 4B).

For frail participants, increased low-intensity PA (type 1) participation (AME=22.48%, 95% CI 1.24-43.72; *P*=.04) and higher SPPB walking subscore (AME=27.73%, 95% CI 4.95%-50.51%; *P*=.02) were associated with an increased probability of improving to robust state. Decreased moderate (type 3; AME=−22.98%, 95% CI −39.17% to −6.79%; *P*=.005) and low-intensity (type 1; AME=−24.02%, 95% CI −46.76% to −1.27%; *P*=.04) PA participation and SPPB walking subscore (AME=−43.68%, 95% CI −75.56% to −11.80%; *P*=.007), along with increased walking speed (AME =157.77%, 95% CI 10.06%-305.49%; *P*=.04), were linked to a higher likelihood of remaining frail (see Figure 4C).

In addition to PA and physical function, several covariates were significantly associated with frailty transitions. Detailed regression results are presented in Tables S1-S3 in Multimedia Appendix 1.

### Sensitivity Analysis

When handgrip strength was removed from the predictor list, the direction and magnitude of AMEs for PA and SPPB subcomponents were unchanged across all baseline frailty states. However, there is one exception: among prefrail participants, the AME for vigorous PA (type 2) on transitioning to robust status attenuated to 20.68% (95% CI −0.23  to  41.60) with *P*=.05, compared to *P*=.04 in the full model. This suggests that the previously observed significance for vigorous PA in prefrail individuals is moderately dependent on concurrent strength measures (see Tables S4–S6 in Multimedia Appendix 1).

### Discriminative Ability of Physical Activity and Physical Function Variables for Frailty Transition

**Figure 5.**
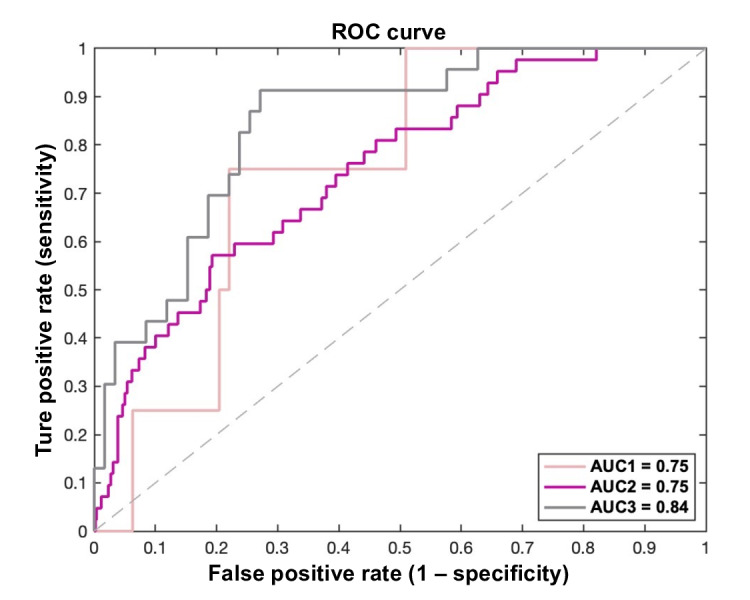
Receiver operating characteristic (ROC) curves for discriminative ability of physical activity and physical function variables. (1) ROC curve 1 (AUC1) for discriminating transition to frailty versus nonfrailty among participants with robust baseline status; (2) ROC curve 2 (AUC2) for discriminating transition to frailty versus nonfrailty among participants with prefrail baseline status. (3) ROC curve 3 (AUC3) for discriminating frailty persistence versus transition to nonfrailty among participants with frail baseline status. AUC: area under the curve.

Figure 5 presents the discriminative performance of PA and physical function variables for frailty transitions across baseline frailty states. Among robust participants, predictors distinguished those who transitioned to frailty from those who remained non-frail with fair accuracy (AUC=0.75, 95% CI 0.53‐0.92; *P*=.04). In the prefrail group, the model similarly discriminated individuals who worsened to frailty from those who remained or improved (AUC=0.75, 95% CI 0.67‐0.82, *P*<.001). Finally, among frail participants, predictors showed good accuracy in identifying those who persisted in frailty versus those who recovered (AUC=0.84, 95% CI 0.75‐0.93; *P*<.001).

## Discussion

### Principal Findings and Comparison With Previous Works

The findings in this study provide valuable insights into how levels of PA participation and physical function measures influence frailty dynamics and highlight how these factors may differ across baseline frailty stages in Chinese older adults. Our results are consistent with the hypothesis that greater amounts of PA and better physical function are associated with a decreased likelihood of worsening frailty or a greater chance of transitioning to a more robust status. This is consistent with previous studies suggesting that PA and physical function are crucial in mitigating frailty progression [13,15]. However, the effect of these factors was not consistent across different baseline frailty states, suggesting that interventions may need to be tailored according to an individual’s frailty stage.

Among participants who were robust at baseline, increased handgrip strength was associated with a higher probability of remaining robust. Conversely, decreased handgrip strength was linked to a higher probability of transitioning to the prefrail stage. Handgrip strength is widely recognized as a reliable indicator of overall muscular strength because it reflects the integrity of the neuromuscular system and correlates strongly with strength in other muscle groups [9,45]. As a simple, noninvasive measure, it provides valuable insights into an individual’s functional capacity and overall health status, making it a practical tool for assessing frailty risk [9,46]. Our finding highlights the importance of maintaining muscular strength in healthy older adults, as it not only helps preserve physical function but also reduces the risk of transitioning to a more frail state [8,9]. In addition, our results suggest that PA level alone did not significantly predict the maintenance or decline in frailty state in this population. It is possible that PA in this group may not have been sufficiently differentiated or intense enough to cause a clear shift in frailty status, as the robust state may represent a relatively stable and resilient group. These findings support that strength-based indicators like handgrip strength may play a more critical role in maintaining or improving frailty status [8,9], indicating that muscle strength could be a more direct determinant of frailty transitions in this group. Strengthening interventions, such as resistance training, could be particularly effective for maintaining robustness in this group and preventing the onset of frailty [47].

For participants who were prefrail at baseline, the findings underscore the importance of vigorous PA and handgrip strength in promoting robustness among prefrail individuals. The amount of vigorous PA per week, which exceeds the recommended PA levels for older adults [48], has been shown to enhance overall health status, and thus for reversing prefrailty [49]. The positive association between vigorous PA and improved frailty status suggests that higher-intensity activities may stimulate physiological adaptations that counteract the decline in physical function associated with aging [50,51]. Conversely, the observed relationships between reduced PA, decreased handgrip strength, and worsening frailty highlight the risks of physical inactivity and muscle loss in this population [52,53]. These results emphasize the need for early and targeted interventions that combine vigorous PA and strength training to mitigate frailty progression for individuals in the prefrail stage. By addressing these modifiable risk factors early in the prefrail stage, it may be possible to delay or even prevent the onset of frailty, ultimately improving the quality of life of older adults and reducing the burden on healthcare systems [54].

In frail participants, increased participation in low-intensity PA and a higher SPPB walking subscore were associated with an increased probability of improving to a robust status. This finding is encouraging, as it suggests that even low-intensity PA can be effective in improving function in frail individuals, potentially reversing frailty and promoting recovery to a more robust state. This aligns with previous studies demonstrating the benefits of low-intensity exercises, such as walking and flexibility training, for frail older adults [55]. Furthermore, decreased participation in moderate and low-intensity PA, along with a lower SPPB walking subscore, was linked to a higher likelihood of remaining frail. This highlights the bidirectional relationship between PA and frailty: while reduced PA increases the risk of frailty, frailty itself can limit PA engagement due to physical limitations. This self-reinforcing cycle of decline, wherein frailty impedes PA participation, has been well-documented in the literature [15,47,56-64]. Our findings further emphasize this dynamic interaction, underscoring the need for early intervention to disrupt this cycle.

Unexpectedly, increased walking speed was associated with a higher likelihood of remaining frail in this study. While counterintuitive, this finding may reflect methodological and physiological nuances specific to the frail cohort. First, the wide CI (10.06%‐305.49%) indicates substantial variability in individual responses, suggesting that small speed improvements in some participants did not translate to holistic functional recovery. Second, frail older adults often experience fluctuations in mobility due to underlying health conditions, which may temporarily elevate walking speed without altering overall frailty status [65-67]. Thus, while improved speed is generally desirable, its isolated increase may be insufficient to reverse multifactorial frailty in this population. Future research should incorporate other mobility measures, such as walking endurance or dual-task gait performance, to better capture functional changes in frail individuals.

Interestingly, only 28% of participants (23/82) classified as frail at baseline remained frail after 2 years, with a recovery rate notably higher than the roughly 50% reported in other longitudinal studies [24]. Several methodological and cohort-specific factors likely contribute to this finding. For instance, selective attrition (ie, exclusion of participants who died or were unable to complete Wave 2) removes the most vulnerable individuals, biasing recovery rates to be upward. In addition, the relatively shorter follow-up period (2 y) and younger age of participants in our study may have contributed to these findings, as improvements in frailty are often more pronounced in younger populations over shorter timescales [24,25]. Finally, reliance on self-reported and occasionally incomplete frailty component data can introduce measurement error, further exaggerating transition rates. Taken together, these factors suggest that the high observed recovery rate may overstate true physiological reversal of frailty and should be interpreted with caution. Future studies with longer follow-up, objective frailty measures, and comprehensive attrition tracking are needed to distinguish genuine recovery from methodological artifacts.

The findings of this study underscore the importance of tailoring interventions based on baseline frailty status. For robust individuals, PA interventions should focus on maintaining strength through resistance training and preventing the decline of muscle function. In prefrail individuals, the emphasis should be on increasing the intensity of PA, especially vigorous activities, and improving handgrip strength to delay or prevent further frailty progression. Our sensitivity analysis confirms that most associations between PA, SPPB subcomponents, and frailty transitions are robust to the exclusion of handgrip strength. The borderline attenuation of the vigorous PA effect among prefrail participants (*P*=.05) indicates a possible interplay between high-intensity activity and muscle strength, warranting further investigation into tailoring PA prescriptions according to baseline strength levels. For frail individuals, interventions should prioritize low-intensity PA and the improvement of basic functional abilities to prevent further deterioration. In addition, factors such as age and gender differences may influence responses to PA interventions, warranting further investigation. Future research with larger sample sizes is needed to explore how personalized interventions can better address the needs of frail individuals by setting achievable goals that gradually build functional capacity without overexertion [68].

This study has several strengths, including its focus on a large cohort of Chinese older adults and the inclusion of both PA and physical function measures. The AUC of discriminative ability for frailty transition capacity is good for participants with a frail state at baseline, suggesting that the model performs well. For individuals with nonfrail status at baseline, the AUC is 0.75, indicating fair discriminative capacity [44]. This is considered meaningful in the context of frailty transitions, which are influenced by a wide range of biological, behavioral, and environmental factors [4].

However, the study also has several limitations. First, the use of self-reported PA data may introduce recall bias, although the IPAQ has been widely used and validated in older populations [37]. Second, the exclusion of participants with missing data may have introduced selection bias. While the CHARLS manual outlined potential reasons for missing data (eg, feeling unsafe or incapable to perform the tests), these reasons were not clearly indicated in the dataset. As a result, it is challenging to accurately determine the cause of missing data for most participants. To avoid speculation, we only proceeded with the available data. This limitation may have contributed to the selection bias as participants appeared healthier at follow-up compared to baseline. Furthermore, our analytic sample included 52% (528/1014) men and 48% (486/1014) women, whereas many older-adult cohort studies report a female majority due to women’s greater longevity [69]. This near-equal sex distribution likely reflects our requirement that participants survive and complete both CHARLS waves (2011 and 2013). Frailer individuals, disproportionately women at advanced ages, were more likely to die or be lost to follow-up, thus attenuating the expected women predominance. We acknowledge this potential selection bias and its implications for generalizability. Third, despite the longitudinal design, this study is observational and cannot establish causal relationships between PA amounts, physical function, and frailty transitions. Longitudinal studies with longer follow-up periods and larger sample sizes could provide further insights into the dynamic nature of frailty and its predictors. Fourth, our findings are based on a specific cohort of Chinese older adults, and the generalizability of these results to other populations remains uncertain. Further studies are needed to explore how these predictors of frailty transitions may vary across different cultural, social, and economic contexts. Finally, our study focused on physical frailty and did not consider other dimensions of frailty, such as cognitive or social frailty, which may also influence frailty transitions. Future research should incorporate a broader range of determinants to provide a more holistic understanding of frailty dynamics.

### Conclusions

In conclusion, this study provides important insights into the role of PA participation and physical function in frailty transitions among older Chinese adults. Our findings suggest that both PA and physical function measures significantly influence frailty dynamics, with varying effects depending on baseline frailty status. Tailored interventions that address the specific needs of robust, prefrail, and frail individuals are essential for preventing or delaying frailty progression. Future research should explore more personalized approaches to PA interventions, especially for frail individuals, to ensure that activities align with their functional capacity and promote meaningful improvement in physical function.

## Supplementary material

10.2196/75887 Multimedia Appendix 1 Ordinal logistic regression analysis of predictors and covariates associated with frailty transitions.

## Abbreviations

ADL: activity of daily living
AME: average marginal effect
AUC: area under the curve
CHARLS: China Health and Retirement Longitudinal Study
CHS: Cardiovascular Health Study
IADL: instrumental activity of daily living
IPAQ: International Physical Activity Questionnaire
MTE: metabolic equivalent
PA: physical activity
PFP: physical frailty phenotype
ROC: receiver operating characteristic
SPPB: Short Physical Performance Battery
STS: sit-to-stand

## References

[1] Chen X, Giles J, Yao Y (2022). The path to healthy ageing in China: a Peking University–Lancet Commission. The Lancet.

[2] Mather M, Kilduff L (2020). Aging and health in China: what can we learn from the world’s largest population of older people. https://www.prb.org/resources/china-aging-worlds-largest-population-older-people/.

[3] Fried LP, Tangen CM, Walston J (2001). Frailty in older adults: evidence for a phenotype. J Gerontol A Biol Sci Med Sci.

[4] Bauer JM, Sieber CC (2008). Sarcopenia and frailty: a clinician’s controversial point of view. Exp Gerontol.

[5] Kojima G, Taniguchi Y, Iliffe S, Jivraj S, Walters K (2019). Transitions between frailty states among community-dwelling older people: A systematic review and meta-analysis. Ageing Res Rev.

[6] He B, Ma Y, Wang C (2019). Prevalence and risk factors for frailty among community-dwelling older people in China: a systematic review and meta-analysis. J Nutr Health Aging.

[7] Op het Veld LPM, van Rossum E, Kempen G, de Vet HCW, Hajema K, Beurskens A (2015). Fried phenotype of frailty: cross-sectional comparison of three frailty stages on various health domains. BMC Geriatr.

[8] Chung CJ, Wu C, Jones M (2014). Reduced handgrip strength as a marker of frailty predicts clinical outcomes in patients with heart failure undergoing ventricular assist device placement. J Card Fail.

[9] Syddall H, Cooper C, Martin F, Briggs R, Aihie Sayer A (2003). Is grip strength a useful single marker of frailty?. Age Ageing.

[10] Cruz-Jentoft AJ, Baeyens JP, Bauer JM (2010). Sarcopenia: European consensus on definition and diagnosis: Report of the European Working Group on Sarcopenia in Older People. Age Ageing.

[11] Verghese J, Xue X (2010). Identifying frailty in high functioning older adults with normal mobility. Age Ageing.

[12] Guralnik JM, Simonsick EM, Ferrucci L (1994). A short physical performance battery assessing lower extremity function: association with self-reported disability and prediction of mortality and nursing home admission. J Gerontol.

[13] Lang PO, Michel JP, Zekry D (2009). Frailty syndrome: a transitional state in a dynamic process. Gerontology.

[14] (2018). Physical activity. World Health Organization.

[15] O’Connell ML, Coppinger T, McCarthy AL (2020). The role of nutrition and physical activity in frailty: A review. Clin Nutr ESPEN.

[16] Pate RR, Pratt M, Blair SN (1995). Physical activity and public health. A recommendation from the Centers for Disease Control and Prevention and the American College of Sports Medicine. JAMA.

[17] Sattelmair J, Pertman J, Ding EL, Kohl HW, Haskell W, Lee IM (2011). Dose response between physical activity and risk of coronary heart disease. Circulation.

[18] Aune D, Norat T, Leitzmann M, Tonstad S, Vatten LJ (2015). Physical activity and the risk of type 2 diabetes: a systematic review and dose–response meta-analysis. Eur J Epidemiol.

[19] Giovannucci E, Ascherio A, Rimm EB, Colditz GA, Stampfer MJ, Willett WC (1995). Physical activity, obesity, and risk for colon cancer and adenoma in men. Ann Intern Med.

[20] Sherrington C, Michaleff ZA, Fairhall N (2017). Exercise to prevent falls in older adults: an updated systematic review and meta-analysis. Br J Sports Med.

[21] Vagetti GC, Barbosa Filho VC, Moreira NB, Oliveira V de, Mazzardo O, Campos W de (2014). Association between physical activity and quality of life in the elderly: a systematic review, 2000-2012. Braz J Psychiatry.

[22] McPhee JS, French DP, Jackson D, Nazroo J, Pendleton N, Degens H (2016). Physical activity in older age: perspectives for healthy ageing and frailty. Biogerontology.

[23] Jia L, Du Y, Chu L (2020). Prevalence, risk factors, and management of dementia and mild cognitive impairment in adults aged 60 years or older in China: a cross-sectional study. Lancet Public Health.

[24] Rivera-Almaraz A, Salinas-Rodríguez A, Gutiérrez-Peña E, Manrique-Espinoza BS (2024). Predictors of frailty transitions in Mexican older adults. J Gerontol A Biol Sci Med Sci.

[25] Rodríguez-Laso Á, García-García FJ, Rodríguez-Mañas L (2022). Transitions Between Frailty States and Its Predictors in a Cohort of Community-Dwelling Spaniards. J Am Med Dir Assoc.

[26] Majid Z, Welch C, Davies J, Jackson T (2020). Global frailty: The role of ethnicity, migration and socioeconomic factors. Maturitas.

[27] Zhao Y, Hu Y, Smith JP, Strauss J, Yang G (2014). Cohort profile: the China Health and Retirement Longitudinal Study (CHARLS). Int J Epidemiol.

[28] Wang R, Chen Z, Zhou Y, Shen L, Zhang Z, Wu X (2019). Melancholy or mahjong? Diversity, frequency, type, and rural-urban divide of social participation and depression in middle- and old-aged Chinese: A fixed-effects analysis. Soc Sci Med.

[29] Lee JSW, Auyeung TW, Leung J, Kwok T, Woo J (2014). Transitions in frailty states among community-living older adults and their associated factors. J Am Med Dir Assoc.

[30] McHugh JE, Dowling M, Butler A, Lawlor BA (2016). Psychological distress and frailty transitions over time in community-dwelling older adults. Ir J Psychol Med.

[31] Wang H, Chen H (2022). Aging in China: challenges and opportunities. China CDC Wkly.

[32] Xu W, Li YX, Hu Y, Wu C (2020). Association of frailty with recovery from disability among community-dwelling Chinese older adults: China health and retirement longitudinal study. BMC Geriatr.

[33] Li Y, Xue QL, Odden MC, Chen X, Wu C (2020). Linking early life risk factors to frailty in old age: evidence from the China Health and Retirement Longitudinal Study. Age Ageing.

[34] Wu C, Smit E, Xue QL, Odden MC (2018). Prevalence and correlates of frailty among community-dwelling Chinese older adults: the China health and retirement longitudinal study. The Journals of Gerontology.

[35] Xu W, Li YX, Wu C (2019). Incidence of frailty among community-dwelling older adults: a nationally representative profile in China. BMC Geriatr.

[36] Sha S, Chan SHW, Chen L, Xu Y, Pan Y (2022). The association between trajectories of loneliness and physical frailty in Chinese older adults: does age matter?. Int J Environ Res Public Health.

[37] Li X, Zhang W, Zhang W (2020). Level of physical activity among middle-aged and older Chinese people: evidence from the China health and retirement longitudinal study. BMC Public Health.

[38] Tian Y, Shi Z (2022). Effects of physical activity on daily physical function in chinese middle-aged and older adults: a longitudinal study from CHARLS. J Clin Med.

[39] Li S, Zhang J, Yang Y (2024). Correlation between the physical activity volume and cognitive and mental capacity among older adult people in China: a cross-sectional study based on the 2020 CHARLS database. Front Public Health.

[40] Zhong BX, Zhong HL, Zhou GQ, Xu WQ, Lu Y, Zhao Q (2021). Physical performance and risk of hip fracture in community-dwelling elderly people in China: A 4-year longitudinal cohort study. Maturitas.

[41] Guralnik JM, Ferrucci L, Simonsick EM, Salive ME, Wallace RB (1995). Lower-extremity function in persons over the age of 70 years as a predictor of subsequent disability. N Engl J Med.

[42] Rubin DB (1987). Multiple Imputation for Survey Nonresponse.

[43] Schmoor C, Schumacher M, Finke J, Beyersmann J (2013). Competing risks and multistate models. Clin Cancer Res.

[44] Swets JA (1988). Measuring the accuracy of diagnostic systems. Science.

[45] Shaughnessy KA, Hackney KJ, Clark BC (2020). A narrative review of handgrip strength and cognitive functioning: bringing a new characteristic to muscle memory. Journal of Alzheimer’s Disease.

[46] Vaishya R, Misra A, Vaish A, Ursino N, D’Ambrosi R (2024). Hand grip strength as a proposed new vital sign of health: a narrative review of evidences. J Health Popul Nutr.

[47] Peterson MJ, Giuliani C, Morey MC (2009). Physical activity as a preventative factor for frailty: the health, aging, and body composition study. J Gerontol A Biol Sci Med Sci.

[48] Piercy KL, Troiano RP, Ballard RM (2018). The physical activity guidelines for Americans. JAMA.

[49] Takamura M, Sone T, Kawamura T, Suzuki R, Moriyama N, Yasumura S (2021). A cross-sectional study on the characteristics of physical activity in pre-frail older adults. Int J Environ Res Public Health.

[50] Kendall KL, Fairman CM (2014). Women and exercise in aging. J Sport Health Sci.

[51] Stamatakis E, Huang BH, Maher C (2021). Untapping the health enhancing potential of vigorous intermittent lifestyle physical activity (VILPA): rationale, scoping review, and a 4-pillar research framework. Sports Med.

[52] Hengeveld LM, Wijnhoven HAH, Olthof MR (2019). Prospective associations of diet quality with incident frailty in older adults: the health, aging, and body composition study. J Am Geriatr Soc.

[53] Doi T, Makizako H, Tsutsumimoto K (2018). Transitional status and modifiable risk of frailty in Japanese older adults: A prospective cohort study. Geriatrics Gerontology Int.

[54] Zeng X zhai, Meng L bing, Li Y ying (2023). Prevalence and factors associated with frailty and pre-frailty in the older adults in China: a national cross-sectional study. Front Public Health.

[55] Tse ACY, Wong TWL, Lee PH (2015). Effect of low-intensity exercise on physical and cognitive health in older adults: a systematic review. Sports Med Open.

[56] Kehler DS, Theou O (2019). The impact of physical activity and sedentary behaviors on frailty levels. Mech Ageing Dev.

[57] Apóstolo J, Cooke R, Bobrowicz-Campos E (2018). Effectiveness of interventions to prevent pre-frailty and frailty progression in older adults: a systematic review. JBI Database System Rev Implement Rep.

[58] Giné-Garriga M, Roqué-Fíguls M, Coll-Planas L, Sitjà-Rabert M, Salvà A (2014). Physical exercise interventions for improving performance-based measures of physical function in community-dwelling, frail older adults: a systematic review and meta-analysis. Arch Phys Med Rehabil.

[59] Angulo J, El Assar M, Álvarez-Bustos A, Rodríguez-Mañas L (2020). Physical activity and exercise: Strategies to manage frailty. Redox Biol.

[60] Savela SL, Koistinen P, Stenholm S (2013). Leisure-time physical activity in midlife is related to old age frailty. J Gerontol A Biol Sci Med Sci.

[61] Borda MG, Pérez-Zepeda MU, Samper-Ternent R, Gómez RC, Avila-Funes JA, Cano-Gutierrez CA (2020). The influence of lifestyle behaviors on the incidence of frailty. J Frailty Aging.

[62] Soares WJS, Lima CA, Bilton TL, Ferrioli E, Dias RC, Perracini MR (2015). Association among measures of mobility-related disability and self-perceived fatigue among older people: a population-based study. Braz J Phys Ther.

[63] Blodgett J, Theou O, Kirkland S, Andreou P, Rockwood K (2015). The association between sedentary behaviour, moderate-vigorous physical activity and frailty in NHANES cohorts. Maturitas.

[64] Stojanovic M, Babulal GM, Head D (2023). Determinants of physical activity engagement in older adults. J Behav Med.

[65] Fried LP, Ferrucci L, Darer J, Williamson JD, Anderson G (2004). Untangling the concepts of disability, frailty, and comorbidity: implications for improved targeting and care. J Gerontol A Biol Sci Med Sci.

[66] Avila-Funes JA, Helmer C, Amieva H (2008). Frailty among community-dwelling elderly people in France: the three-city study. J Gerontol A Biol Sci Med Sci.

[67] Cowper PA, Peterson MJ, Pieper CF (2017). Economic analysis of primary care-based physical activity counseling in older men: the VA-LIFE Trial. J Am Geriatr Soc.

[68] Allison R, Assadzandi S, Adelman M (2021). Frailty: evaluation and management. Am Fam Physician.

[69] Zeidan RS, McElroy T, Rathor L, Martenson MS, Lin Y, Mankowski RT (2023). Sex differences in frailty among older adults. Exp Gerontol.

