# Supplementary material for: The Role of Physical Activity and Physical Function in Predicting Physical Frailty Transitions in Chinese Older Adults: Longitudinal Observational Study From CHARLS
Source: JMIR Aging. 2025 Sep 15;8:e75887. doi: 10.2196/75887 (PMC12443357; doi:10.2196/75887)
Supplement: Multimedia Appendix 1 [file aging-v8-e75887-s001.docx]

Table S1. Ordinal logistic regression analysis of predictors and covariates that associated with frailty transitions among participants with robust baseline status.

|  | Coefficient (*β*) | SE | 95% CI | *P*-value |
| --- | --- | --- | --- | --- |
| Vigorous PA (1) | 0.99 | 0.91 | -0.79 to 2.77 | .28 |
| Vigorous PA (2) | -0.31 | 0.48 | -1.26 to 0.63 | .52 |
| Vigorous PA (3) | -0.58 | 0.31 | -1.20 to 0.03 | .06 |
| Moderate PA (1) | 0.07 | 0.52 | -0.95 to 1.09 | .89 |
| Moderate PA (2) | 0.15 | 0.32 | -0.48 to 0.78 | .64 |
| Moderate PA (3) | -0.21 | 0.32 | -0.85 to 0.42 | .51 |
| Low-intensity PA (1) | 0.08 | 0.57 | -1.03 to 1.19 | .89 |
| Low-intensity PA (2) | 0.18 | 0.51 | -0.82 to 1.17 | .73 |
| Low-intensity PA (3) | 0.30 | 0.52 | -0.72 to 1.33 | .56 |
| SPPB (1) | -0.41 | 0.78 | -1.94 to 1.13 | .60 |
| SPPB (2) | -0.69 | 1.09 | -2.83 to 1.46 | .53 |
| SPPB-Walk | 0.39 | 0.32 | -0.24 to 1.02 | .22 |
| SPPB-STS | -0.26 | 0.30 | -0.84 to 0.32 | .39 |
| SPPB-Balance | 0.31 | 0.25 | -0.18 to 0.79 | .22 |
| Handgrip strength | -0.06 | 0.02 | -0.10 to -0.01 | **.02** |
| Walking speed | -1.55 | 1.22 | -3.94 to 0.83 | .20 |
| STS time | -0.02 | 0.04 | -0.10 to 0.07 | .69 |
| Age | 0.08 | 0.03 | 0.02 to 0.13 | **.01** |
| Gender (Female) | -1.18 | 0.46 | -2.08 to -0.28 | **.01** |
| BMI | 0.02 | 0.03 | -0.05 to 0.09 | .56 |
| Marital status (Married) | 0.52 | 0.32 | -0.11 to 1.14 | .10 |
| Education level (2) | -0.05 | 0.37 | -0.78 to 0.68 | .90 |
| Education level (3) | -0.02 | 0.33 | -0.68 to 0.63 | .94 |
| Education level (4) | -0.71 | 0.38 | -1.45 to 0.03 | .06 |
| Self-rated health (1) | -0.49 | 0.29 | -1.07 to 0.08 | .09 |
| Fall history (1) | -0.09 | 0.34 | -0.75 to 0.58 | .80 |
| ADL limitation type (1) | -0.16 | 0.36 | -0.86 to 0.55 | .67 |
| IADL limitation type (1) | 0.30 | 0.33 | -0.34 to 0.94 | .36 |
| Number of chronic diseases (1) | 0.76 | 0.32 | 0.13 to 1.38 | **.02** |
| Number of chronic diseases (2) | 0.44 | 0.31 | -0.17 to 1.06 | .16 |
| Pain (1) | 0.72 | 0.32 | 0.10 to 1.34 | **.02** |
| Current smoking (1) | -0.30 | 0.33 | -0.96 to 0.35 | .37 |
| Current drinking (1) | -0.46 | 0.28 | -1.01 to 0.08 | .10 |

*Notes: ADL Activity of daily living; BMI Body mass index; IADL Instrumental activity of daily living; PA Physical activity; SE Standard Error; SPPB Short Physical Performance Battery; STS Sit-To-Stand; CI Confidence interval.* *Boldface indicates statistical significance (P<.05).*

Table S2. Ordinal logistic regression analysis of predictors and covariates that associated with frailty transitions among participants with pre-frail baseline status.

|  | Coefficient (*β*) | SE | 95% CI | *P*-value |
| --- | --- | --- | --- | --- |
| Vigorous PA (1) | 0.14 | 0.91 | -1.64 to 1.92 | .88 |
| Vigorous PA (2) | -1.11 | 0.51 | -2.11 to -0.12 | **.03** |
| Vigorous PA (3) | -0.34 | 0.24 | -0.82 to 0.13 | .16 |
| Moderate PA (1) | -0.34 | 0.44 | -1.20 to 0.52 | .44 |
| Moderate PA (2) | -0.01 | 0.24 | -0.48 to 0.48 | .98 |
| Moderate PA (3) | -0.18 | 0.27 | -0.71 to 0.35 | .51 |
| Low-intensity PA (1) | -0.37 | 0.32 | -1.00 to 0.26 | .25 |
| Low-intensity PA (2) | -0.31 | 0.26 | -0.83 to 0.21 | .24 |
| Low-intensity PA (3) | -0.08 | 0.30 | -0.66 to 0.50 | .79 |
| SPPB (1) | -0.59 | 0.41 | -1.40 to 0.23 | .16 |
| SPPB (2) | -0.48 | 0.68 | -1.80 to 0.85 | .48 |
| SPPB-Walk | -0.19 | 0.25 | -0.68 to 0.30 | .45 |
| SPPB-STS | -0.14 | 0.16 | -0.45 to 0.17 | .39 |
| SPPB-Balance | -0.18 | 0.16 | -0.50 to 0.14 | .27 |
| Handgrip strength | -0.04 | 0.01 | -0.06 to -0.01 | **.004** |
| Walking speed | 0.19 | 0.97 | -1.71 to 2.10 | .84 |
| STS time | -0.03 | 0.03 | -0.08 to 0.03 | .32 |
| Age | 0.05 | 0.02 | 0.01 to 0.10 | **.03** |
| Gender (Female) | -0.70 | 0.29 | -1.28 to -0.13 | **.02** |
| BMI | -0.04 | 0.03 | -0.10 to 0.02 | .19 |
| Marital status (Married) | -0.06 | 0.22 | -0.48 to 0.37 | .80 |
| Education level (2) | 0.06 | 0.26 | -0.44 to 0.57 | .81 |
| Education level (3) | -0.03 | 0.25 | -0.52 to 0.46 | .90 |
| Education level (4) | -0.83 | 0.31 | -1.43 to -0.23 | **.007** |
| Self-rated health (1) | -0.13 | 0.28 | -0.68 to 0.43 | .66 |
| Fall history (1) | 0.52 | 0.24 | 0.05 to 0.99 | **.03** |
| ADL limitation type (1) | 0.23 | 0.23 | -0.23 to 0.69 | .32 |
| IADL limitation type (1) | 0.36 | 0.23 | -0.09 to 0.81 | .12 |
| Number of chronic diseases (1) | 0.38 | 0.27 | -0.14 to 0.91 | .15 |
| Number of chronic diseases (2) | 0.53 | 0.26 | 0.03 to 1.03 | **.04** |
| Pain (1) | 0.43 | 0.22 | -0.01 to 0.86 | .05 |
| Current smoking (1) | 0.25 | 0.27 | -0.28 to 0.77 | .36 |
| Current drinking (1) | -0.56 | 0.23 | -1.02 to -0.11 | **.02** |

*Notes: ADL Activity of daily living; BMI Body mass index; IADL Instrumental activity of daily living; PA Physical activity; SE Standard Error; SPPB Short Physical Performance Battery; STS Sit-To-Stand; CI Confidence interval. Boldface indicates statistical significance (P<.05).*

Table S3. Ordinal logistic regression analysis of predictors and covariates that associated with frailty transitions among participants with frail baseline status.

|  | Coefficient (*β*) | SE | 95% CI | *P*-value |
| --- | --- | --- | --- | --- |
| Vigorous PA (2) | -0.05 | 1.33 | -2.66 to 2.56 | .97 |
| Vigorous PA (3) | -0.65 | 1.45 | -3.50 to 2.19 | .65 |
| Moderate PA (1) | 1.30 | 1.61 | -1.85 to 4.46 | .42 |
| Moderate PA (2) | 0.66 | 0.99 | -1.27 to 2.60 | .50 |
| Moderate PA (3) | -2.29 | 1.28 | -4.80 to 0.23 | .08 |
| Low-intensity PA (1) | -2.13 | 1.08 | -4.24 to -0.03 | **.047** |
| Low-intensity PA (2) | -1.57 | 1.04 | -3.61 to 0.47 | .13 |
| Low-intensity PA (3) | 0.27 | 1.32 | -2.33 to 2.87 | .84 |
| SPPB (1) | 1.50 | 1.08 | -0.61 to 3.61 | .16 |
| SPPB (2) | 1.39 | 2.48 | -3.46 to 6.24 | .58 |
| SPPB-Walk | -3.30 | 1.24 | -5.73 to -0.87 | **.008** |
| SPPB-STS | 0.23 | 0.49 | -0.73 to 1.19 | .64 |
| SPPB-Balance | -0.50 | 0.41 | -1.31 to 0.32 | .23 |
| Handgrip strength | 0.03 | 0.06 | -0.09 to 0.15 | .63 |
| Walking speed | 11.93 | 5.97 | 0.23 to 23.62 | **.046** |
| STS time | 0.04 | 0.11 | -0.18 to 0.25 | .73 |
| Age | 0.09 | 0.07 | -0.04 to 0.22 | .16 |
| Gender (Female) | -0.47 | 1.17 | -2.77 to 1.83 | .69 |
| BMI | -0.09 | 0.09 | -0.26 to 0.08 | .30 |
| Marital status (Married) | -0.41 | 0.86 | -2.10 to 1.27 | .63 |
| Education level (2) | -0.40 | 1.16 | -2.67 to 1.88 | .73 |
| Education level (3) | -1.43 | 1.05 | -3.49 to 0.63 | .17 |
| Education level (4) | -0.26 | 0.89 | -2.01 to 1.49 | .77 |
| Self-rated health (1) | -0.60 | 1.32 | -3.19 to 1.98 | .65 |
| Fall history (1) | -0.01 | 0.69 | -1.37 to 1.35 | .99 |
| ADL limitation type (1) | 1.54 | 0.77 | 0.02 to 3.05 | **.047** |
| IADL limitation type (1) | 0.19 | 0.70 | -1.18 to 1.56 | .79 |
| Number of chronic diseases (1) | 0.02 | 1.05 | -2.04 to 2.08 | .99 |
| Number of chronic diseases (2) | 1.28 | 1.03 | -0.74 to 3.30 | .22 |
| Pain (1) | 0.50 | 0.62 | -0.71 to 1.72 | .42 |
| Current smoking (1) | 0.64 | 0.81 | -0.94 to 2.23 | .43 |
| Current drinking (1) | -0.57 | 0.94 | -2.41 to 1.26 | .54 |

*Notes: ADL Activity of daily living; BMI Body mass index; IADL Instrumental activity of daily living; PA Physical activity; SE Standard Error; SPPB Short Physical Performance Battery; STS Sit-To-Stand; CI Confidence interval. Boldface indicates statistical significance (P<.05).*

Table S4. Ordinal logistic regression analysis of predictors and covariates that associated with frailty transitions among participants with robust baseline status (sensitivity analysis excluding handgrip strength).

|  | Coefficient (*β*) | SE | 95% CI | *P*-value |
| --- | --- | --- | --- | --- |
| Vigorous PA (1) | 1.06 | 0.85 | -0.61 to 2.74 | .21 |
| Vigorous PA (2) | -0.32 | 0.47 | -1.24 to 0.60 | .49 |
| Vigorous PA (3) | -0.51 | 0.31 | -1.12 to 0.10 | .10 |
| Moderate PA (1) | 0.06 | 0.53 | -0.97 to 1.09 | .91 |
| Moderate PA (2) | 0.07 | 0.31 | -0.54 to 0.69 | .82 |
| Moderate PA (3) | -0.27 | 0.32 | -0.89 to 0.36 | .41 |
| Low-intensity PA (1) | 0.06 | 0.56 | -1.03 to 1.15 | .92 |
| Low-intensity PA (2) | 0.23 | 0.50 | -0.74 to 1.21 | .64 |
| Low-intensity PA (3) | 0.35 | 0.51 | -0.65 to 1.35 | .49 |
| SPPB (1) | -0.53 | 0.77 | -2.03 to 0.98 | .49 |
| SPPB (2) | -0.92 | 1.07 | -3.02 to 1.18 | .39 |
| SPPB-Walk | 0.43 | 0.32 | -0.20 to 1.05 | .18 |
| SPPB-STS | -0.22 | 0.28 | -0.78 to 0.33 | .43 |
| SPPB-Balance | 0.35 | 0.24 | -0.13 to 0.82 | .16 |
| Walking speed | -1.64 | 1.20 | -3.99 to 0.71 | .17 |
| STS time | -0.01 | 0.04 | -0.09 to 0.07 | .82 |
| Age | 0.08 | 0.03 | 0.02 to 0.14 | **.007** |
| Gender (Female) | -0.55 | 0.36 | -1.25 to 0.15 | .12 |
| BMI | < 0.01 | 0.03 | -0.06 to 0.07 | .94 |
| Marital status (Married) | 0.55 | 0.32 | -0.07 to 1.17 | .08 |
| Education level (2) | -0.05 | 0.36 | -0.76 to 0.66 | .89 |
| Education level (3) | -0.02 | 0.33 | -0.67 to 0.63 | .96 |
| Education level (4) | -0.83 | 0.37 | -1.56 to -0.10 | **.03** |
| Self-rated health (1) | -0.56 | 0.29 | -1.13 to 0.02 | .06 |
| Fall history (1) | -0.06 | 0.35 | -0.74 to 0.62 | .87 |
| ADL limitation type (1) | -0.16 | 0.35 | -0.84 to 0.52 | .65 |
| IADL limitation type (1) | 0.35 | 0.32 | -0.28 to 0.99 | .28 |
| Number of chronic diseases (1) | 0.66 | 0.32 | 0.04 to 1.27 | **.04** |
| Number of chronic diseases (2) | 0.41 | 0.31 | -0.19 to 1.02 | .18 |
| Pain (1) | 0.66 | 0.31 | 0.05 to 1.27 | **.03** |
| Current smoking (1) | -0.23 | 0.34 | -0.89 to 0.43 | .50 |
| Current drinking (1) | -0.50 | 0.28 | -1.06 to 0.06 | .08 |

*Notes: ADL Activity of daily living; BMI Body mass index; IADL Instrumental activity of daily living; PA Physical activity; SE Standard Error; SPPB Short Physical Performance Battery; STS Sit-To-Stand; CI Confidence interval.* *Boldface indicates statistical significance (P<.05).*

Table S5. Ordinal logistic regression analysis of predictors and covariates that associated with frailty transitions among participants with pre-frail baseline status (sensitivity analysis excluding handgrip strength).

|  | Coefficient (*β*) | SE | 95% CI | *P*-value |
| --- | --- | --- | --- | --- |
| Vigorous PA (1) | 0.32 | 1.00 | -1.63 to 2.27 | .75 |
| Vigorous PA (2) | -1.05 | 0.51 | -2.05 to -0.05 | **.04** |
| Vigorous PA (3) | -0.32 | 0.24 | -0.80 to 0.16 | .19 |
| Moderate PA (1) | -0.35 | 0.44 | -1.21 to 0.51 | .42 |
| Moderate PA (2) | -0.02 | 0.25 | -0.50 to 0.47 | .95 |
| Moderate PA (3) | -0.22 | 0.27 | -0.75 to 0.31 | .42 |
| Low-intensity PA (1) | -0.29 | 0.31 | -0.91 to 0.32 | .35 |
| Low-intensity PA (2) | -0.23 | 0.26 | -0.74 to 0.29 | .39 |
| Low-intensity PA (3) | 0.04 | 0.29 | -0.53 to 0.61 | .88 |
| SPPB (1) | -0.50 | 0.41 | -1.31 to 0.31 | .23 |
| SPPB (2) | -0.35 | 0.68 | -1.68 to 0.97 | .60 |
| SPPB-Walk | -0.18 | 0.26 | -0.68 to 0.32 | .47 |
| SPPB-STS | -0.16 | 0.16 | -0.47 to 0.15 | .32 |
| SPPB-Balance | -0.21 | 0.16 | -0.52 to 0.11 | .20 |
| Walking speed | 0.19 | 1.00 | -1.78 to 2.16 | .85 |
| STS time | -0.02 | 0.03 | -0.07 to 0.03 | .39 |
| Age | 0.05 | 0.02 | 0.02 to 0.11 | **.006** |
| Gender (Female) | -0.35 | 0.28 | -0.89 to 0.19 | .21 |
| BMI | -0.05 | 0.03 | -0.11 to 0.01 | .09 |
| Marital status (Married) | -0.10 | 0.22 | -0.52 to 0.33 | .65 |
| Education level (2) | 0.03 | 0.26 | -0.48 to 0.53 | .92 |
| Education level (3) | -0.07 | 0.25 | -0.55 to 0.42 | .78 |
| Education level (4) | -0.93 | 0.31 | -1.53 to -0.33 | **.002** |
| Self-rated health (1) | -0.16 | 0.28 | -0.72 to 0.40 | .58 |
| Fall history (1) | 0.52 | 0.24 | 0.05 to 0.99 | **.03** |
| ADL limitation type (1) | 0.24 | 0.23 | -0.22 to 0.70 | .31 |
| IADL limitation type (1) | 0.36 | 0.23 | -0.09 to 0.81 | .12 |
| Number of chronic diseases (1) | 0.38 | 0.27 | -0.14 to 0.91 | .15 |
| Number of chronic diseases (2) | 0.53 | 0.25 | 0.03 to 1.03 | **.04** |
| Pain (1) | 0.37 | 0.22 | -0.05 to 0.79 | .09 |
| Current smoking (1) | 0.23 | 0.26 | -0.29 to 0.75 | .39 |
| Current drinking (1) | -0.53 | 0.23 | -0.98 to -0.08 | **.02** |

*Notes: ADL Activity of daily living; BMI Body mass index; IADL Instrumental activity of daily living; PA Physical activity; SE Standard Error; SPPB Short Physical Performance Battery; STS Sit-To-Stand; CI Confidence interval. Boldface indicates statistical significance (P<.05).*

Table S6. Ordinal logistic regression analysis of predictors and covariates that associated with frailty transitions among participants with frail baseline status (sensitivity analysis excluding handgrip strength).

|  | Coefficient (*β*) | SE | 95% CI | *P*-value |
| --- | --- | --- | --- | --- |
| Vigorous PA (2) | -0.01 | 1.30 | -2.56 to 2.53 | .99 |
| Vigorous PA (3) | -0.63 | 1.42 | -3.41 to 2.16 | .66 |
| Moderate PA (1) | 1.42 | 1.52 | -1.56 to 4.39 | .35 |
| Moderate PA (2) | 0.59 | 0.97 | -1.31 to 2.49 | .54 |
| Moderate PA (3) | -2.22 | 1.22 | -4.62 to 0.18 | .07 |
| Low-intensity PA (1) | -2.09 | 1.08 | -4.22 to 0.03 | .05 |
| Low-intensity PA (2) | -1.49 | 1.07 | -3.59 to 0.60 | .16 |
| Low-intensity PA (3) | 0.23 | 1.31 | -2.33 to 2.80 | .86 |
| SPPB (1) | 1.39 | 1.10 | -0.76 to 3.54 | .21 |
| SPPB (2) | 1.43 | 2.58 | -3.63 to 6.49 | .58 |
| SPPB-Walk | -3.15 | 1.11 | -5.33 to -0.98 | **.005** |
| SPPB-STS | 0.26 | 0.50 | -0.71 to 1.23 | .61 |
| SPPB-Balance | -0.47 | 0.42 | -1.30 to 0.35 | .26 |
| Walking speed | 11.50 | 5.61 | 0.50 to 22.49 | **.04** |
| STS time | 0.04 | 0.11 | -0.18 to 0.25 | .73 |
| Age | 0.09 | 0.07 | -0.04 to 0.22 | .17 |
| Gender (Female) | -0.58 | 1.07 | -2.68 to 1.53 | .59 |
| BMI | -0.08 | 0.09 | -0.26 to 0.09 | .34 |
| Marital status (Married) | -0.32 | 0.86 | -2.01 to 1.36 | .71 |
| Education level (2) | -0.32 | 1.16 | -2.59 to 1.96 | .78 |
| Education level (3) | -1.37 | 1.16 | -3.63 to 0.90 | .24 |
| Education level (4) | -0.13 | 0.90 | -1.89 to 1.64 | .89 |
| Self-rated health (1) | -0.44 | 1.31 | -3.02 to 2.13 | .74 |
| Fall history (1) | 0.06 | 0.65 | -1.21 to 1.33 | .92 |
| ADL limitation type (1) | 1.63 | 0.78 | 0.11 to 3.16 | **.04** |
| IADL limitation type (1) | 0.17 | 0.70 | -1.20 to 1.54 | .81 |
| Number of chronic diseases (1) | 0.10 | 1.01 | -1.87 to 2.07 | .92 |
| Number of chronic diseases (2) | 1.24 | 1.04 | -0.79 to 3.27 | .23 |
| Pain (1) | 0.50 | 0.61 | -0.70 to 1.70 | .42 |
| Current smoking (1) | 0.78 | 0.80 | -0.78 to 2.35 | .33 |
| Current drinking (1) | -0.64 | 0.90 | -2.40 to 1.11 | .47 |

*Notes: ADL Activity of daily living; BMI Body mass index; IADL Instrumental activity of daily living; PA Physical activity; SE Standard Error; SPPB Short Physical Performance Battery; STS Sit-To-Stand; CI Confidence interval. Boldface indicates statistical significance (P<.05).*
